# Supplementary material for: Hybrid Models and Biological Model Reduction with PyDSTool
Source: PLoS Comput Biol. 2012 Aug 9;8(8):e1002628. doi: 10.1371/journal.pcbi.1002628 (PMC3415397; doi:10.1371/journal.pcbi.1002628)
Supplement: Text S4 — Complete source code for the PyDSTool package (version 0.88.120504). Includes API documentation and help files linking to web pages. This file is identical to the current public release on Sourceforge.net. (ZIP) [file pcbi.1002628.s004.zip › PyDSTool/html/PyDSTool.Toolbox.event_driven_simulator-pysrc.html]

xml version="1.0" encoding="ascii"?


PyDSTool.Toolbox.event\_driven\_simulator


| Home | Trees | Indices | Help | PyDSTool |
| --- | --- | --- | --- | --- |

Package PyDSTool ::
Package Toolbox ::
Module event\_driven\_simulator

[hide private]

[frames] | no frames]

# Source Code for Module PyDSTool.Toolbox.event\_driven\_simulator

```
  1  from PyDSTool.common import * 
  2  from PyDSTool.errors import * 
  3  from PyDSTool.utils import remain, info 
  4  from PyDSTool.Points import Point, Pointset 
  5  from numpy import array, asarray, NaN, Inf, isfinite 
  6  from PyDSTool import figure, plot 
  7  from pylab import gca 
  8  from copy import copy 
  9   
 10  _classes = ['connection', 'node', 'simulator', 'composed_map1D', 
 11              'map', 'map1D', 'map2D', 'identity_map', 'delay_map'] 
 12   
 13  _functions = ['extract_digraph'] 
 14   
 15  _instances = ['idmap'] 
 16   
 17  __all__ = _classes + _functions + _instances 
 18   
 19  # ------------------------------------------------------------------------------ 
 20   


21 -class connection(object):


22 -    def __init__(self, name):


23          self.name = name 
 24          # input is a node object 
 25          self.input = None 
 26          # output is for informational purposes only 
 27          # (nodes keyed by name) 
 28          self.outputs = {} 
 29          # map is of type map (defaults to identity) 
 30          self.map = idmap

 31   


32 -    def poll(self):


33          # poll input 
 34          return self.map(self.input.last_t)

 35   


36 -    def __repr__(self):


37          return "connection(%s)" % self.name

 38   
 39      __str__ = __repr__

 40   
 41   


42 -class node(object):


43 -    def __init__(self, name):


44          self.name = name 
 45          # inputs are connection objects 
 46          self.inputs = {} 
 47          # output is for informational purposes only 
 48          # (connections keyed by name) 
 49          self.outputs = {} 
 50          # projected state 
 51          self.next_t = 0 
 52          # last state (set by simulator.set_state) 
 53          self.last_t = 0 
 54          # current input values 
 55          self.in_vals = {} 
 56          # map attribute will be of type map 
 57          self.map = None

 58   


59 -    def poll(self, state):


60          #print "\n ** ", self.name, "received state:", state 
 61          self.in_vals.update(state) 
 62          # poll inputs 
 63          for name, connxn in self.inputs.items(): 
 64              self.in_vals[connxn.input.name] = connxn.poll() 
 65          #print "... passing input values", self.in_vals 
 66          self.next_t = self.map(self.in_vals) 
 67          return self.next_t

 68   


69 -    def __repr__(self):


70          return "node(%s)" % self.name

 71   
 72      __str__ = __repr__

 73   
 74   


75 -class FIFOqueue_uniquenode(object):


76      """Only one entry per node is allowed. 
 77      !! Does not allow for simultaneous events !! 
 78      """ 


79 -    def __init__(self, node_names):


80          self.nodes = node_names 
 81          self.reset()

 82   


83 -    def push(self, t, node_name):


84          old_t = self.by_node[node_name] 
 85          if t != old_t: 
 86              self.by_node[node_name] = t 
 87              self.by_time[t] = node_name 
 88              if isfinite(old_t): 
 89                  del self.by_time[old_t] 
 90              self.next_t = min(self.by_time.keys())

 91   


92 -    def reset(self):


93          self.by_time = {Inf: None} 
 94          self.next_t = Inf 
 95          self.by_node = dict.fromkeys(self.nodes, Inf)

 96   


97 -    def pop(self):


98          t = self.next_t 
 99          if isfinite(t): 
100              val = (t, self.by_time[t]) 
101              del self.by_time[t] 
102              self.by_node[val[1]] = Inf 
103              self.next_t = min(self.by_time.keys()) 
104              return val 
105          else: 
106              raise PyDSTool_UndefinedError("Empty queue")

107   
108   


109 -def extract_digraph(mspec, node_types, connxn_types):


110      """Extract directed graph of connections from a ModelSpec description 
111      of a dynamical systems model, using the node and connection types 
112      provided. The name attributes of those types are used to search the 
113      ModelSpec. 
114      """ 
115      connxn_names = mspec.search(connxn_types) 
116      node_names = mspec.search(node_types) 
117   
118      # declare connection and node objects 
119      connxns = {} 
120      nodes = {} 
121      for c in connxn_names: 
122          cobj = mspec.components[c] 
123          new_connxn = connection(c) 
124          connxns[c] = new_connxn 
125   
126      for n in node_names: 
127          nobj = mspec.components[n] 
128          new_node = node(n) 
129          nodes[n] = new_node 
130   
131      # fill in inputs and outputs dictionaries for each type 
132      for cn, c in connxns.items(): 
133          cobj = mspec.components[cn] 
134          targs = [head(t) for t in cobj.connxnTargets] 
135          c.outputs = dict(zip(targs, [nodes[t] for t in targs])) 
136          for t in targs: 
137              nodes[t].inputs[cn] = c 
138   
139      for nn, n in nodes.items(): 
140          nobj = mspec.components[nn] 
141          targs = [head(t) for t in nobj.connxnTargets] 
142          n.outputs = dict(zip(targs, [connxns[t] for t in targs])) 
143          for t in targs: 
144              connxns[t].input = n 
145   
146      return nodes, connxns

147   
148   


149 -def head(hier_name):


150      if '.' in hier_name: 
151          return hier_name.split('.')[0] 
152      else: 
153          return hier_name

154   


155 -def tail(hier_name):


156      if '.' in hier_name: 
157          return hier_name.split('.')[-1] 
158      else: 
159          return hier_name

160   
161  # maps' inputs and output are absolute times -- 
162  # for dealing with relative times they must be passed a reference 
163  # time value to add to a relative time. 


164 -class map(object):


165      pass

166   


167 -class map2D(map):


168      pass

169   


170 -class map1D(map):


171      pass

172   
173   


174 -class delay_map(map1D):


175 -    def __init__(self, delay):


176          self.delay = delay

177   


178 -    def __call__(self, t):


179          return t + self.delay

180   
181   


182 -class identity_map(map1D):


183 -    def __call__(self, t):


184          return t

185   
186   
187  # default instance of identity class 
188  idmap = identity_map() 
189   
190   


191 -class composed_map1D(map1D):


192 -    def __init__(self, m1, m2):


193          self.m1 = m1 
194          self.m2 = m2

195   


196 -    def __call__(self, t):


197          return self.m1(self.m2(t))

198   
199  # ----------------------------------------------------------------------------- 
200   


201 -class simulator(object):


202      """Mapping-based, event-driven simulator for 
203      dynamical systems reductions. 
204      """ 


205 -    def __init__(self, nodes, connections, state=None):


206          self.nodes = nodes 
207          # don't really need the connections, but just as a reference 
208          self.connections = connections 
209          if state is None: 
210              self.state = dict(zip(nodes.keys(), [NaN for n in nodes])) 
211          else: 
212              self.state = state 
213          self.curr_t = 0 
214          self.history = {} 
215          self.verbosity = 0 
216          self.Q = FIFOqueue_uniquenode(nodes.keys())

217   


218 -    def set_node_state(self):


219          for name, n in self.nodes.items(): 
220              n.last_t = self.state[name]

221   


222 -    def validate(self):


223          for name, n in self.nodes.items(): 
224              assert isinstance(n, node) 
225          for name, connxn in self.connections.items(): 
226              assert isinstance(connxn, connection) 
227          assert sortedDictKeys(self.state) == sortedDictKeys(self.nodes), \ 
228                 "Invalid or missing node state in history argument"

229   


230 -    def run(self, history, t_end):


231          """history is a dictionary of t -> (event node name, {node name: state}) 
232          values. Initial time of simulator will be the largest t in this 
233          dictionary. 
234          """ 
235          self.curr_t = max(history.keys()) 
236          assert t_end > self.curr_t, "t_end too small" 
237          self.state = history[self.curr_t][1].copy() 
238   
239          # structural validation of model 
240          self.validate() 
241          node_names = sortedDictKeys(self.state) 
242          self.history = history.copy() 
243          done = False 
244   
245          while not done: 
246              last_state = self.state.copy() 
247              print "\n ***", self.curr_t, self.history[self.curr_t][0], self.state 
248   
249              next_t = Inf 
250              iters = 1 
251              nodes = self.nodes.copy() 
252              # set the last_t of each node 
253              self.set_node_state() 
254   
255              try: 
256                  proj_state = self.compile_next_state(nodes) 
257              except PyDSTool_BoundsError: 
258                  print "Maps borked at", self.curr_t 
259                  done = True 
260                  break 
261              print "Projected:", proj_state 
262              for node, t in proj_state.items(): 
263                  if t > self.curr_t: 
264                      self.Q.push(t, node) 
265  #            self.state[next_node] = self.Q.next_t 
266   
267              if self.verbosity > 0: 
268                  print "Took %i iterations to stabilize" % iters 
269                  self.display() 
270   
271              t, next_node = self.Q.pop() 
272              while self.curr_t > t: 
273                  t, next_node = self.Q.pop() 
274              self.curr_t = t 
275              self.history[self.curr_t] = (next_node, last_state) 
276              ##print " * last state =", last_state 
277              next_state = last_state 
278              next_state[next_node] = self.curr_t 
279              # must copy here to ensure history elements don't get 
280              # overwritten 
281              self.state = next_state.copy() 
282   
283              if self.verbosity > 0: 
284                  print "Next node is", next_node, "at time ", self.curr_t 
285              done = self.curr_t >= t_end 
286              continue 
287   
288              ## 
289   
290              vals = sortedDictValues(projected_states) 
291              filt_vals = [] 
292              min_val = Inf 
293              min_ix = None 
294              for i, v in enumerate(vals): 
295                  if v > self.curr_t: 
296                      if v < min_val: 
297                          min_val = v 
298                          min_ix = i 
299  ##                else: 
300  ##                    # do not ignore projected times that are in the past relative to 
301  ##                    # curr_t! Must retrgrade curr_t to the earliest newly projected time. 
302  ##                    if v not in self.history: 
303  ##                        if v < min_val: 
304  ##                            min_val = v 
305  ##                            min_ix = i 
306              if min_ix is None: 
307                  # no further events possible 
308                  print "No further events possible, stopping!" 
309                  break 
310  ##            if min_val < self.curr_t: 
311  ##                # clear later history 
312  ##                print "Clearing later history items that are invalid" 
313  ##                for t, s in sortedDictItems(self.history): 
314  ##                    if t > min_val: 
315  ##                        del self.history[t] 
316              self.curr_t = min_val 
317              next_state = self.state[1].copy() 
318              next_node = node_names[min_ix] 
319              next_state[next_node] = min_val 
320              self.history[min_val] = (next_node, next_state) 
321              self.state = (next_node, next_state) 
322   
323              if self.verbosity > 0: 
324                  print "Next node is", next_node, "at time ", self.curr_t 
325              done = self.curr_t >= t_end 
326   
327          ts, state_dicts = sortedDictLists(self.history, byvalue=False) 
328          vals = [] 
329          for (evnode, vd) in state_dicts: 
330              vals.append([vd[nname] for nname in node_names]) 
331          self.result = Pointset(indepvararray=ts, indepvarname='t', 
332                                 coordnames = node_names, 
333                                 coordarray = array(vals).T)

334   
335   


336 -    def compile_next_state(self, nodes):


337          vals = {} 
338          for name, n in nodes.items(): 
339              vals[name] = n.poll(self.state) 
340          return vals

341   


342 -    def display(self):


343          print "\n****** t =", self.curr_t 
344          info(self.state, "known state") 
345          print "\nNodes:" 
346          for name, n in self.nodes.items(): 
347              #n.poll(self.state) 
348              print name 
349              for in_name, in_val in n.in_vals.items(): 
350                  print "  Input", in_name, ": ", in_val

351   


352 -    def extract_history_events(self):


353          node_names = self.nodes.keys() 
354          node_events = dict(zip(node_names, [None]*len(node_names))) 
355          ts = sortedDictKeys(self.history) 
356          old_node, old_state = self.history[ts[0]] 
357          # deal with initial conditions first 
358          for nn in node_names: 
359              node_events[nn] = [old_state[nn]] 
360          # do the rest 
361          for t in ts: 
362              node, state = self.history[t] 
363              node_events[node].append(t) 
364          return node_events

365   


366 -    def display_raster(self, new_figure=True):


367          h = self.history 
368          ts = sortedDictKeys(h) 
369          node_names = self.nodes.keys() 
370          print "\n\nNode order in plot (bottom to top) is", node_names 
371          if new_figure: 
372              figure() 
373          t0 = ts[0] 
374          node, state = h[t0] 
375          # show all initial conditions 
376          for ni, n in enumerate(node_names): 
377              plot(state[n], ni, 'ko') 
378          # plot the rest 
379          for t in ts: 
380              node, state = h[t] 
381              plot(t, node_names.index(node), 'ko') 
382          a = gca() 
383          a.set_ylim(-0.5, len(node_names)-0.5)

384   
385   


386 -def sequences_to_eventlist(seq_dict):


387      """seq_dict maps string symbols to increasing-ordered sequences of times. 
388      Returns a single list of (symbol, time) pairs ordered by time.""" 
389      out_seq = [] 
390      symbs = seq_dict.keys() 
391      next_s = None 
392      indices = {} 
393      for s in symbs: 
394          indices[s] = 0 
395      remaining_symbs = symbs 
396      while remaining_symbs != []: 
397          #print "\n***", remaining_symbs 
398          to_remove = [] 
399          #print indices 
400          #print out_seq, "\n" 
401          next_t = Inf 
402          for s in remaining_symbs: 
403              try: 
404                  t = seq_dict[s][indices[s]] 
405              except IndexError: 
406                  # no times remaining for this symbol 
407                  #print "No more symbols for ", s 
408                  to_remove.append(s) 
409              else: 
410                  #print s, t 
411                  if t < next_t: 
412                      next_s = s 
413                      next_t = t 
414                      #print "Chose ", next_s, t 
415          indices[next_s] += 1 
416          for s in to_remove: 
417              remaining_symbs.remove(s) 
418          if isfinite(next_t): 
419              out_seq.append( (next_s, next_t) ) 
420      return out_seq

421   
422  # --------------------------------------------------------------------- 
423   
424  if __name__ == '__main__': 


425 -    class testmap1D(map1D):


426 -        def __init__(self, targ, val):


427              self.targ = targ 
428              self.val = val

429   


430 -        def __call__(self, state):


431              return self.val + state[self.targ]

432   


433 -    class testmap2D(map2D):


434 -        def __init__(self, targ, me):


435              self.targ = targ 
436              self.me = me

437   


438 -        def __call__(self, state):


439              return 3 + state[self.targ] + 0.1*state[self.me]

440   
441   
442      xmap = testmap1D('x', 2) 
443      ymap = testmap2D('y', 'x') 
444      x = node('x') 
445      y = node('y') 
446   
447      xcy = connection('xcy') 
448      xcy.input = x 
449      xcy.outputs = {'y': y} 
450   
451      ycx = connection('ycx') 
452      ycx.input = y 
453      ycx.map = delay_map(2) 
454      ycx.outputs = {'x': x} 
455   
456      xcx = connection('xcx') 
457      xcx.input = x 
458      xcx.outputs = {'x': x} 
459   
460      x.inputs['y'] = ycx 
461      x.inputs['x'] = xcx 
462      x.map = ymap 
463      y.inputs['x'] = xcy 
464      y.map = xmap 
465   
466      sim = simulator({'x': x, 'y': y}, {'xcy': xcy, 'ycx': ycx, 
467                                         'xcx': xcx}) 
468      sim.verbosity = 1 
469      sim.run({0: ('x', {'x': -5, 'y': -2}), -2: ('y', {'x': -5, 'y': -8})}, 10) 
470
```

  


| Home | Trees | Indices | Help | PyDSTool |
| --- | --- | --- | --- | --- |

|  |  |
| --- | --- |
| Generated by Epydoc 3.0.1 on Fri May 4 15:24:19 2012 | http://epydoc.sourceforge.net |
